# ASO Practice Guidelines Series: Resectable Colorectal Cancer

**DOI:** 10.1245/s10434-026-20216-z

**Published:** 2026-07-27

**Authors:** Ramy Behman, George J. Chang

**Affiliations:** University of Texas MD Anderson Cancer Center - Colon and Rectal Surgery, Houston, TX USA

**Keywords:** Colon cancer, Rectal cancer, Practice guidelines, Total neoadjuvant therapy, Mismatch repair deficiency

## Abstract

Colorectal cancer remains a leading cause of cancer morbidity and mortality worldwide. Despite biologic similarities, colon and rectal cancers require distinct management strategies. Rapid advances molecular characterization (including mismatch repair status) as well as paradigm shifts in neoadjuvant therapy and organ-preservation strategies have expanded the range of treatment approaches while increasing the need for clear, guideline-anchored decision-making. This Practice Guidelines Series article summarizes contemporary guidance from major international guidelines (National Comprehensive Cancer Network (NCCN), European Society for Medical Oncology (ESMO), and Japanese Society for Cancer of the Colon and Rectum (JSCCR)), highlighting areas of recent evolution and ongoing debate.

Colorectal cancer is the third most common cancer and the second leading cause of cancer-related deaths worldwide, accounting for approximately 10% of all new cancer diagnoses and 9.4% of global cancer deaths.^1,2^ Despite many similarities, colon and rectal cancer represent distinct diseases with differing management approaches. Herein we provide a summary of practice guidelines for the management of colon and rectal cancers in separate subsections. Each subsection focuses on areas of recent developments and debate and highlights differences and similarities between different international guidelines (National comprehensive cancer network (NCCN), European society for medical oncology (ESMO), and Japanese society for cancer of the colon and rectum (JSCCR)).

## Colon Cancer

### Staging

Following histologic confirmation of colon cancer, accurate clinical staging is essential to inform treatment planning. Cross-sectional imaging with contrast-enhanced computed tomography (CT) of the chest, abdomen, and pelvis remains the management standard for initial staging per NCCN and ESMO recommendations.^3,4^ Positron emission tomography (PET)/CT is not indicated for routine staging but may be appropriate in equivocal cases or when metastasectomy is being considered.^5^ Baseline serum carcinoembryonic antigen (CEA) measurement is recommended both for prognosis and subsequent surveillance.^6^ Additionally, all patients should undergo complete colonoscopy to exclude synchronous neoplasms, which occur in approximately 3% of cases.^7^ If the primary tumor is obstructing and prevents full visualization, this limitation should be documented and completion colonoscopy performed within 6 months after resection.

Clinical staging is based primarily on imaging (e.g., contrast enhanced CT of the chest, abdomen, and pelvis or noncontrast enhanced CT of the chest and magnetic resonance imaging (MRI) of the abdomen and pelvis), while definitive staging requires pathologic evaluation of the resection specimen. The limitations of CT for tumor (T) and node (N) classification are well recognized; prospective trials randomizing patients on the basis of preoperative imaging have demonstrated over-staging in nearly one-quarter to one-third of cases.^8–10^

Final staging requires excision and microscopic evaluation of tumor depth, lymph node metastasis, and presence of tumor deposits. All guidelines stress that at least 12 lymph nodes should be examined in the surgical specimen for proper pathologic staging. Inadequate nodal evaluation (< 12 nodes) is considered a high-risk feature in stage II disease due to the risk of under-staging. Additional adverse features including lymphovascular or perineural invasion, tumor budding, obstruction, perforation, and poor differentiation (excluding microsatellite instability-high (MSI-H)) are also recognized as markers of higher recurrence risk and should be included in pathologic staging as they may guide adjuvant therapy decisions.

While not formally part of staging, molecular characterization is now an integral component of initial evaluation. Deficient mismatch repair (dMMR) or MSI-high status is present in 10–15% of colon cancers and should be assessed universally, as endorsed by all major guidelines. This has implications for Lynch syndrome testing, adjuvant systemic therapy decisions, and eligibility for immunotherapy. dMMR tumors do not respond favorably to fluoropyrimidine monotherapy but are highly sensitive to checkpoint inhibitors. Detection of dMMR/MSI-H tumors should prompt additional testing for BRAF, and if negative, hypermethylation of MLH1. BRAF mutation or MLH1 hypermethylation is consistent with a sporadic tumor, whereas negative results should prompt additional testing to rule out Lynch syndrome. Molecular profiling (e.g., RAS and BRAF mutations, HER2 amplification, PI3K pathway) has generally been recommended in metastatic disease as it may inform systemic therapy options. The NCCN only recently began recommending molecular profiling on all stage II and III colon cancers, as patients with PI3K pathway alterations would benefit from the addition of aspirin concurrently with their adjuvant therapy (regardless of MSI status). Similarly, circulating tumor DNA (ctDNA) is not currently part of staging, but has been shown to be an important predictor of recurrence, with potential implications for guiding adjuvant therapy.

### Neoadjuvant Therapy

Traditionally, resectable colon cancer has been managed with upfront surgery. However, emerging evidence has prompted incorporation of neoadjuvant systemic therapy in selected patients.

### Neoadjuvant Therapy in pMMR (Mismatch-Repair Proficient) Disease

For mismatch repair proficient (pMMR) or microsatellite stable disease, neoadjuvant chemotherapy is considered for bulky nodal involvement or cT4b tumors at risk for incomplete resection. The FOxTROT trial randomized more than 1000 patients with operable CT-staged T3–T4 colon cancers to 3 cycles of preoperative FOLFOX followed by 9 cycles postoperatively versus 12 cycles of adjuvant therapy alone. Preoperative treatment was delivered safely in 98% of patients, achieved significant tumor regression, increased R0 resection rates (94% versus 89%, *p* < 0.001), and reduced early recurrence at 2 years (16.9% versus 21.5%, hazard ratio 0.72, *p* = 0.037). Overall survival did not differ, but these data support the potential benefit of tumor downstaging and early systemic control.^10^ Additional trials such as OPTICAL and NEOCOL confirmed improved tolerance and down-staging, though survival results were mixed.^8,9^ A persistent challenge across studies has been CT-based over-staging, observed in 24–33% of patients assigned to adjuvant-only arms. NCCN accordingly issues a category 2A recommendation allowing individualized use of preoperative oxaliplatin-based chemotherapy in pMMR patients with cT4 or bulky nodal disease. ESMO guidelines reserve neoadjuvant chemotherapy for investigational settings or technically unresectable tumors. The JSCCR continues to endorse upfront surgery for resectable cases.^11^

### Neoadjuvant Therapy in dMMR Disease

In contrast, for dMMR or MSI-high colon cancers, chemotherapy provides little benefit in the neoadjuvant setting. These tumors are highly immunogenic, and recent studies have demonstrated dramatic responses to checkpoint inhibitors. The NICHE-1 study first showed that dual checkpoint blockade with nivolumab and ipilimumab produced substantial pathologic responses, particularly in MSI-H tumors.^12^ Building on this, NICHE-2 enrolled 112 patients with nonmetastatic dMMR colon cancer and administered two doses of dual immunotherapy prior to surgery; 95% achieved major pathologic response, including 68% percent with complete pathologic response, and nearly all exhibited tumor regression. Toxicity was modest, with only 4% developing significant immune-related adverse events, and almost all patients underwent timely resection. At more than 2 years of follow-up, no recurrences were observed.^13^ These promising outcomes have been incorporated into NCCN guidance, which now recommends neoadjuvant checkpoint blockade as the preferred strategy in MSI-H locally advanced colon cancer. ESMO and JSCCR have not yet adopted this approach outside clinical trials, although widespread use is anticipated.

Many centers would now favor neoadjuvant immunotherapy in patients with MSI-H, and high-risk colon cancer and studies are currently underway exploring the role of definitive immunotherapy followed by a watch and wait approach to this subset of colon cancer, although the challenges of endoscopic tumor response assessment for more proximal colon tumors must be considered.

### Surgical Principles for Resectable Colon Cancer

#### Extent of Bowel Resection

Surgery remains the cornerstone of curative treatment. Resection should encompass the involved colonic segment with adequate proximal and distal margins and complete lymphadenectomy of the nodal basin.

The extent of bowel resection should be determined by the anatomic distribution of the primary feeding vessel to ensure complete resection of the tumor and its associated lymphatics. In addition, to ensure resection of potential areas of intramural spread, both NCCN and ESMO recommend gross margins of at least 5 cm. However, Japanese anatomic studies suggest that margins of 10 cm are needed to capture all longitudinal spread to pericolic lymph nodes.

Negative radial margins are especially important for locally advanced tumors that extend into the retroperitoneum and sites lacking peritoneal covering. A recent US national study observed an 11.6% rate of radial margin involvement after colon cancer surgery, which increased to 31.5% for T4b tumors.^14^ Involved adjacent structures or organs should therefore be removed en bloc to ensure complete resection. As mentioned previously, patients with cT4b disease and/or those with suspected threatened margins may be considered for neoadjuvant chemotherapy, as the FOxTROT trial demonstrated significantly improved R0 resection rates in these patients.^15^

### Principles of Radical Lymphadenectomy

Lymph node metastasis is an important factor affecting both treatment and prognosis in cancer. Complete en bloc resection of the regional lymph node basin draining the primary cancer is a core principle of surgical treatment. The lymphadenectomy should be guided by the primary blood supply to the tumor bearing segment and proximal ligation of the named feeding vessel(s) should be performed. When the tumor location falls between two primary vessels, both should be included in the resection (Table 1).

**Table 1:** Primary feeding vessels by colon tumor location

Primary tumor location	Primary feeding vessel(s)
Cecum	Ileocolic artery, right colic artery*
Ascending colon	Ileocolic artery Right colic artery* Right branch of the middle colic artery**
Hepatic flexure	Ileocolic artery Right colic artery* Right branch of the middle colic artery
Transverse colon	Middle colic artery
Splenic flexure	Left branch of the middle colic artery Left colic artery
Descending colon	Inferior mesenteric artery
Sigmoid colon	Superior rectal artery
Rectosigmoid	Superior rectal artery

*When present

**When right colic artery absent or tumor between right colic artery and middle colic artery

### Extent of Lymphadenopathy

Lymphadenectomy is a critical component of oncologic colon resection, both for staging and for potential therapeutic benefit. Because lymphatic spread from colon cancer typically follows the vascular arcade from pericolic to intermediate and then central nodes, the extent of nodal dissection has been formalized by the JSCCR into graded levels (D1, D2, and D3, respectively), and in Western practice, as complete mesocolic excision (CME) with emphasis on the integrity and completeness of the investing fascia of the mesocolon, paired with central vascular ligation. The rationale underpinning these approaches is to achieve complete resection of disease and accurate staging or optimal assignment of adjuvant chemotherapy and to improve local control and survival.

### D2 versus D3 versus CME

A D2 dissection removes pericolic and intermediate nodes to the root of the supplying vessel, whereas a D3/CME lymphadenectomy extends the dissection along the central vessels to clear central nodes. There is considerable overlap in the definitions of CME and D3 lymphadenectomy, with both emphasizing resection of the central nodes. Historically, CME has been described as including central vascular ligation (CVL) with sharp dissection in the embryologic mesocolic plane to remove the tumor and its draining lymphovascular network as an intact specimen, although these are often required for a D3 lymphadenectomy as well.

In Japan, the JSCCR guidelines have historically recommended a D3 dissection for invasive colon cancers beyond very early lesions. This approach has been standard in Japan for decades and is based on their data showing up to 10.4% of patients with metastases at central nodes even without intermediate nodes involved (skip metastases).^16^ Notably, data from Japan have also shown that 10% of pT1 colon cancers have lymph node metastases, with 2% having spread to intermediate nodes, suggesting that all but the lowest risk invasive colon cancers require at least D2 lymphadenectomy. cT2 tumors have a 1% chance of metastasis to central nodes.^17^

In Western practice, surgeons often use the term CME with CVL, which emphasizes sharp dissection along embryologic planes for resection with high vascular tie and closely parallels a D3 lymphadenectomy. CME involves removal of an intact packet of mesocolon containing the tumor and lymphatics, analogous to the total mesorectal excision (TME) concept in rectal cancer. Hohenberger’s classic series showed that meticulous CME surgery improved local recurrence and survival compared with historical controls.^18^ Studies comparing CME with high-quality D2 lymphadenectomy have yielded mixed results. European studies have reported that CME yields more lymph nodes and possibly better oncologic outcomes than non-CME surgery.^19–21^ For example, a Danish population-based study reported improved 4-year disease-free survival with CME versus conventional resection (85.8% versus 75.9% for stage I–III patients).^22^ However, the RELARC trial, which was a randomized trial comparing D2 lymphadenectomy versus CME in right-sided colon cancer, showed no significant difference in disease-free or overall survival.^23^ Taken together, CME with D3 resections for colon cancer have not been universally accepted as the standard of care, in part due to technical difficulty and concerns for vascular injury during dissection.

However, in the modern era of surgery for colon cancer, a complete D2 lymphadenectomy (resection to the origin of the feeding vessel) with an intact mesocolon should be considered the minimum. While NCCN does not explicitly use the terms D2/D3 or CME in its recommendations, it suggests that clinically involved central nodes should also be removed if possible, and mandates retrieval of ≥ 12 nodes for sufficient resection. ESMO guidelines similarly emphasize that surgery for colon cancer should follow oncologic principles with high vascular tie and mesocolic plane dissection. JSCCR, in contrast, is prescriptive about the exact dissection level based on T stage (e.g., D2 for T1, D3 for ≥ T2, or any clinical nodal involvement).^17^

### Management of Malignant Polyps (T1 Cancers)

The management of T1 cancers resected endoscopically is informed by histopathologic features. Tumors with low-risk features, including well or moderately differentiated histology, absence of lymphovascular invasion, negative resection margins of at least 1–2 mm, and limited submucosal invasion (Haggitt levels 1–3 for pedunculated lesions or Kikuchi sm1 for sessile lesions), may be observed after endoscopic removal. The variability in the definition of negative resection margins (no-ink-on-tumor, 1 mm, 2 mm, etc.) between societies and guidelines highlights the fact that the impact of close margins is not well understood. Pathologic findings of any high-risk features including poor differentiation, tumor budding, lymphovascular invasion, close or positive margins, and deep submucosal invasion (Haggitt 4 or Kikuchi sm2 and sm3, > 1000 μm) warrant formal colectomy with lymphadenectomy. Residual disease or nodal metastasis risk in the presence of adverse features ranges from 10% to 25%. Both NCCN and ESMO endorse this risk-stratified approach. In all settings, en bloc resection should be prioritized as piecemeal removal precludes accurate pathologic assessment. Generally, in settings of piecemeal resection in which the presence of high-risk features cannot be excluded, patients should be fully staged and undergo oncologic surgical resection. At present, NCCN guidelines advocate for oncologic resection for patients with piecemeal resection and indeterminate margins. In practice, highly selected patients may be considered for short-interval endoscopic reevaluation, although it should be noted that there is no guidance on surveillance for this patient population.

It is worth noting that very low-risk T1 cancers that have been completely removed endoscopically (see malignant polyps below) might avoid surgery altogether. However, if surgery is performed for a malignant polyp or T1 cancer, at least D2 (including pericolic and intermediate nodes) is recommended, since up to ~10% of T1 have nodal metastases.^17^ All surgeries should ensure simultaneous resection of any suspicious lymph nodes outside the standard field if encountered.

### Adjuvant Therapy

Adjuvant systemic therapy decisions are determined on the basis of risk for recurrence, which correlate with stage-specific prognosis. In patients with node negative disease (stage II), for whom prognosis is generally favorable, chemotherapy is considered for patients with other high-risk features such as T4, poor differentiation, lymphovascular or perineural invasion, obstruction or perforation, close margins, or inadequate nodal harvest per NCCN guidelines. The absolute survival benefit is modest, generally in the range of 3–5%. ESMO guidelines stratify stage II into low, medium, and high risk, with treatment recommended for the highest-risk group. dMMR node-negative tumors derive no benefit from fluoropyrimidines and are usually observed, given their favorable prognosis.

Patients with node positive disease or presence of tumor deposits (stage III), are recommended to undergo adjuvant chemotherapy in all but the frailest patients. Oxaliplatin-based regimens such as FOLFOX or CAPOX are standard. Historically, 6 months of therapy was prescribed, but the IDEA collaboration, pooling more than 12,000 patients, demonstrated that 3 months of CAPOX was noninferior to 6 months for low-risk stage III (T1–3, N1) with substantially reduced neurotoxicity.^24^ In addition, 3 months of FOLFOX was not noninferior to 6 months, and thus CAPOX is favored for shorter duration regimens. The shortened duration of adjuvant chemotherapy was associated with a significant decrease in the incidence of grade 3–4 peripheral neuropathy from 15.9% to 2.5% for those treated with FOLFOX and from 8.9% to 2.6% for those treated with CAPOX. High-risk stage III (T4 or N2) continues to require 6 months of therapy. NCCN, ESMO, and JSCCR now endorse a risk-adapted duration: 3 months of CAPOX for low-risk disease and 6 months of oxaliplatin-based therapy for high-risk disease. Adjuvant therapy should ideally commence within 6 weeks postoperatively, as delays beyond 6 weeks reduce efficacy.^25^

### Deescalation Strategies

Recent years have seen a movement toward deescalation in colorectal cancer treatment, reducing treatment intensity, side effects, and morbidity, while maintaining oncologic outcomes.

Circulating tumor DNA (ctDNA) is increasingly being utilized as an adjunct to help inform decision-making about adjuvant chemotherapy, particularly in the high-risk stage II group of patients. Though not yet in guidelines, research shows that stage II patients who are ctDNA-positive after surgery have a very high recurrence risk and might benefit most from chemotherapy, whereas ctDNA-negative patients could safely forego adjuvant therapy.^26^ More recent trials (e.g., NRG-GI005 COBRA in the USA, and DYNAMIC in Australia) have tested ctDNA-guided adjuvant therapy to guide adjuvant therapy. Both studies explored the role for ctDNA for treatment intensification. The COBRA trial showed no improvement in ctDNA clearance with chemotherapy following resection of stage II colon cancer without high-risk features, suggesting there may not be a role for ctDNA-guided intensification in this patient population, but this study was limited by the sensitivity of the assay.^27,28^ Similarly, the DYNAMIC trial showed no benefit in recurrence-free survival with ctDNA-guided treatment intensification in stage III colon cancer.^29^ It should be noted that at present, ctDNA-guided treatment (either escalation or deescalation) should only be performed as part of a clinical trial, and that NCCN guidelines are clear that there is insufficient evidence at present for the use of ctDNA to guide treatment outside of a clinical trial.

As mentioned previously, definitive immunotherapy followed by a watch and wait approach for complete responders is under investigation in the dMMR subset of patients with colon cancer. In NICHE-2, 68% had pathologic complete response, but all patients still underwent surgery. For rectal cancer, nonoperative management (“watch and wait”) after complete response to neoadjuvant therapy is an accepted option in experienced centers. For colon cancer, this has not become standard but is currently offered within clinical trials.

## Rectal Cancer

Rectal cancer is often defined as a tumor with its lower border within approximately 15 cm of the anal verge, though other definitions also exist (including relationships to the sacral promontory, anterior peritoneal reflection, rectosigmoid junction, etc.). The peritoneal reflection is a particularly important anatomic landmark in defining the rectum, as the bowel proximal to this is covered by serosa and felt to behave more like colon cancer with respect to the risk of locoregional spread and recurrence. Distal to the peritoneal reflection, the rectum is entirely non-peritonealized, resulting in higher risk for radial margin involvement with locally advanced tumors. Tumors below the peritoneal reflection also have distinct patterns of lymphatic drainage and tumor spread, through the lymphatic channels that accompany the middle rectal vessels, which may drain into the internal iliac and obturator lymph node basins.

Rectal cancer is a complex malignancy that requires a multidisciplinary treatment approach. Advances such as total mesorectal excision (TME) surgery and neoadjuvant therapy have dramatically improved local control and survival rates. Modern management of rectal cancer requires precise clinical staging, multidisciplinary evaluation, tailored neoadjuvant strategies, and meticulous surgical technique.

### Staging

Accurate clinical staging begins with endoscopic confirmation and pathologic assessment, and universally includes rectal-protocoled pelvic MRI together with systemic staging by CT. Similar to colon cancer, PET is not routinely indicated. MRI is preferred for local staging because it delineates the mesorectal fascia; evaluates tumor depth, nodal involvement, and circumferential resection margin proximity; and identifies adverse features such as extramural vascular invasion and enlarged mesorectal and lateral pelvic nodes that stratify patients into distinct risk pathways.^30,31^ Early lesions can be adjunctively staged with endorectal ultrasound to define depth of invasion to distinguish between T1/T2,^32^ although the accuracy of endoscopic ultrasound is highly user dependent.

All patients with rectal adenocarcinoma should undergo biopsy with histologic confirmation and testing for mismatch repair (MMR) status. Deficient mismatch repair (dMMR) or microsatellite instability-high (MSI-H) tumors comprise roughly 2.3–6.7% of rectal cancers, a lower incidence than seen in colon cancers.^30,33–35^ dMMR rectal cancers exhibit similar reduced chemosensitivity and excellent response to immunotherapy as that seen in colon cancer.^30^

### Neoadjuvant Therapy

Neoadjuvant therapy has historically been considered in the management of stage II–III rectal cancer. The goals of neoadjuvant therapy are to decrease risk for recurrence, improve resectability, sterilize margins, and treat microscopic spread. The decision-making regarding the components and sequence of neoadjuvant therapy are complex and often tailored to the individual patient. The approach can be broadly divided by tumor MMR status, given the emerging role of immunotherapy for dMMR tumors.

### Neoadjuvant Approaches in MMR-Proficient Rectal Cancer

For pMMR stage II–III disease, traditional long-course chemoradiation (lcCRT), typically 50.4 Gy with concurrent fluoropyrimidine, is associated with 5 year pelvic recurrence rates of about 5% and pathologic complete response (pCR) in 10–30% of patients.^36–38^ Historically, lcCRT has been followed by surgery after an 8- to 12-week interval to maximize tumor regression. Short-course radiotherapy (scRT), 25 Gy in five fractions, is an alternative to lcCRT. It has seen greater adoption in Europe than in the USA and is historically administered with a short interval to surgery, therefore without opportunity for downstaging. scRT offers logistical convenience with no difference in the rate of local control and shorter time to full-dose systemic treatment (e.g., in the stage IV setting).^39^

Historically, adjuvant chemotherapy has been administered following radiotherapy and surgery. More recently, the total neoadjuvant therapy (TNT) strategy, in which all intended chemotherapy and radiation is administered prior to surgery, has become increasingly utilized and is now the recommended approach in the NCCN guidelines. TNT can be accomplished via different sequencing: (1) induction chemotherapy followed by chemoradiation, or (2) (chemo)radiation first, then consolidation chemotherapy, before surgery, or (3) as chemotherapy alone with selective chemoradiotherapy. The rationale for TNT is to treat systemic micrometastatic disease early, improve treatment response rates, and ensure patients receive full-dose systemic therapy.

Two landmark phase 3 randomized trials, RAPIDO and PRODIGE 23, demonstrated the benefits of TNT over the traditional approach of adjuvant chemotherapy. In the RAPIDO trial, patients with high-risk locally advanced rectal cancer received scRT followed by 18 weeks of chemotherapy prior to surgery, versus standard chemoradiation and surgery. TNT significantly reduced the 3-year rate of disease-related treatment failure (23.7% versus 30.4%) and significantly lowered the risk of distant metastases (approximately 20% versus 27% at 3 years).^40^ However, there was no difference in overall survival between the study arms, and the 5-year follow-up data from the RAPIDO trial showed a higher risk of local recurrence in the scRT TNT arm compared with lcCRT.^41^

The French PRODIGE-23 trial (FOLFIRINOX induction chemotherapy and lcCRT versus lcCRT) reported higher a pCR rate (28% versus 12%) and improved 3-year disease-free survival with TNT.^42^ The long-term follow-up also showed improved overall survival with TNT (7-year OS 81.9% versus 76.1%) compared with neoadjuvant chemoradiation alone.^43^

Guidelines now endorse TNT as a preferred option for patients with stage III or “high-risk” stage II rectal cancers, particularly those with features such as threatened margins or extensive nodal disease. The components and sequence of TNT can be tailored to patient fitness and tumor characteristics such as location, threatened margins, and clinical nodal status and EMVI that may reflect local versus systemic failure risk.

Another important recent development is the selective use of neoadjuvant chemotherapy alone (omitting radiation) in low- and intermediate-risk rectal cancers. The phase 3 PROSPECT trial tested an omission of routine radiation in moderate-risk rectal cancer: patients with stage II/III tumors of the mid-to-upper rectum (excluding T4 or threatened CRM cases) were randomized to receive either standard chemoradiation versus neoadjuvant FOLFOX chemotherapy for 12 weeks, with chemoradiation reserved only for those who did not respond to chemotherapy with at least 20% clinical reduction in tumor size. After 5-year follow-up, PROSPECT showed that neoadjuvant chemotherapy-first was noninferior to chemoradiation in terms of disease-free survival and local control.^44^ Similarly, no significant difference in outcomes were observed with FOLFOX versus FOLFOX-CRT versus 5-FU-CRT in the FOWARC phase 3 trial.^45^ Avoiding radiation in these cases can spare patients the potential long-term toxicities of pelvic irradiation (such as sexual dysfunction, infertility, and bowel dysfunction).

### Tailoring the Neoadjuvant Approach

The recommendations from different guidelines are summarized in Table 2. However, the neoadjuvant approach should be individualized on the basis of tumor characteristics and patient factors.

**Table 2:** Comparison of neoadjuvant therapy guidelines in locally advanced (stage II–III) rectal cancer

Guideline	NCCN (2024)	ESMO (2022)	JSCCR (2024)
Use of neoadjuvant therapy	Recommends neoadjuvant treatment for virtually all stage II–III rectal cancers.	Recommends neoadjuvant treatment for most stage II–III. Advises risk-adapted use with primary surgery considered for low-risk tumors.	Recommends surgery-first for resectable stage II–III in standard practice. Preoperative therapy is selectively utilized, generally reserved for cases where downsizing is needed (e.g., threatened margin or very low tumor)
Chemoradiation versus short-course RT	lcCRT or scRT, although favors lcCRT in high-risk cases given higher local recurrence risk in RAPIDO 5-year follow-up. scRT remains an option, especially if a shorter schedule is needed.	lcCRT or scRT, although favors lcCRT in high-risk cases given higher local recurrence risk in RAPIDO 5-year follow-up. scRT remains an option, especially if a shorter schedule is needed.	Endorses long-course chemoradiation when preoperative therapy is used.
Total neoadjuvant therapy (TNT)	TNT is the preferred approach and for stage T3+, N any, and T any N+, with the exception of non-T4 eligible for sphincter preserving surgery (i.e., PROSPECT cohort), who may receive chemotherapy alone if there is adequate response on restaging. Non-prescriptive with regards to sequencing.	TNT recommended for most locally advanced cases. Explicitly recommends TNT for patients with high-risk features and all cT2N+ or cT3+ tumors. Non-prescriptive with regards to sequencing.	Recommends against routine use of TNT outside of clinical trials.

The preferred approach within the authors’ unit considers the risk of local versus distant risk progression as well as tumor location and tailors treatment on the basis of these factors. In all cases, decision-making is made in a multidisciplinary fashion. For tumors located in the distal rectum that would require APR or those that have threatened margins on MRI but are without other high-risk features, treat with radiation as the first line of treatment, followed by chemotherapy to consolidate response. In the absence of distant metastasis, lcCRT chemoradiation is preferred to scRT in light of the long-term local recurrence risk noted in the RAPIDO cohort. Alternatively, for tumors in which distant failure is of greater concern (those with EMVI, extensive nodal involvement including lateral pelvic lymph nodes, or tumor deposits), treat with a TNT approach that begins with induction FOLFIRINOX chemotherapy followed by radiotherapy to address systemic risk upfront and improve survival. Tumors in the mid/upper rectum that are N+ but with clear margins may be treated with chemotherapy alone, with selective omission of radiation therapy per the PROSPECT trial. Tumors with favorable features (e.g. cT3, N0, clear CRM) might be adequately managed with upfront surgery.

### Organ Preservation with “Watch and Wait”

Organ preservation strategies are an evolving aspect of tailoring therapy. In patients with a clinical complete response to neoadjuvant therapy (no visible tumor on endoscopy and MRI, and no palpable tumor on exam), a “watch and wait” approach with deferral of surgery may be considered. Multiple guidelines acknowledge that for stage II–III patients who attain a clinical complete response (cCR), especially those unfit for surgery or strongly wishing to avoid a low rectal resection, a watch and wait strategy under strict surveillance can be an option.

Prospective studies and registry data (e.g., the International Watch & Wait Database) indicate that about 20–30% of such patients can avoid surgery initially, though approximately one-third may experience local tumor regrowth within 2–3 years and require salvage TME surgery. Oncologic outcomes (distant metastasis and survival) appear similar between an initial watch and wait with salvage approach versus immediate surgery in highly selected complete responders, provided that any regrowth is detected early and resected. However, this approach should be limited to experienced centers with strict follow-up protocols, and patients must be fully informed of recurrence risks.

### Neoadjuvant Approaches in dMMR Rectal Cancer

The management of dMMR/MSI-H rectal cancer is undergoing a paradigm shift. These tumors are comparatively chemo-resistant to fluoropyrimidines yet demonstrate profound sensitivity to PD-1 blockade. A phase II study first reported a 100% clinical complete response in 12 patients treated with neoadjuvant single-agent immunotherapy.^46^ An expanded cohort presented subsequently confirmed a 100% cCR in 49 patients and recurrence-free survival at 2 years was 96%.^47^ No patients in the trial required escalation to chemoradiation and the incidence of grade 3 adverse event rates was 4.3%. Despite being nonrandomized, these results have dramatically altered the management of patients with dMMR rectal cancers. Patients with dMMR should be triaged to centers offering immunotherapy trials whenever possible, and multidisciplinary teams should recognize that fluoropyrimidine monotherapy has limited value in this biology. When a complete clinical response without stricture follows immunotherapy, the watch and wait approach with close surveillance is emerging as the preferred approach. Routine MSI/MMR testing at diagnosis is therefore recommended to direct eligibility for immunotherapy-based strategies as updates to formal guidelines continue to evolve.

The role of immunotherapy in patients with Lynch syndrome and the possibility of metachronous colorectal cancer is an evolving field.

### Surgical Principles

#### Total Mesorectal Excision

Surgery remains the definitive local therapy, with total mesorectal excision the central operative principle. For upper rectal tumors, a tumor-specific mesorectal excision extending 5 cm distal to the tumor may suffice, whereas mid- and low-rectal tumors require a total mesorectal excision down to the pelvic floor. Execution of a proper TME requires adherence to anatomical planes, requiring identification of the mesorectal fascia and maintaining dissection outside this envelope, thus preserving the mesorectum intact. The specimen should be cylindrical with a smooth, shiny surface when completed. Pathology assessment of the resected specimen’s mesorectal grade (complete versus near complete versus incomplete) correlates with outcomes: an incomplete mesorectal excision (with defects of the mesorectal fat and visible muscle) is associated with higher local recurrence. Local and distant recurrence rates are similar for patients with complete and near-complete mesocolic excision. Specimen quality correlates with outcomes and high-quality TME reduces local recurrence from pre-TME rates of 20–30% to below 10%.^48,49^

### Nerve Preservation

The pelvic autonomic nerves, including the superior hypogastric plexus, hypogastric nerves, and pelvic splanchnic nerves (inferior hypogastric plexus), run in proximity to the mesorectum and can be injured during rectal resection or lymph node dissection. These nerves control urinary continence, sexual function (erectile function in men, vaginal lubrication in women), and bowel motility. Unnecessary damage leads to complications such as urinary retention, impotence/sexual dysfunction, or altered bowel habits. The hypogastric trunk is located posteriorly near the sacral promontory and then divides to run posterolaterally along both sides of the pelvis and careful posterior dissection can avoid injury. The pelvic splanchnic nerves are in the lateral pelvis; avoidance of excessive lateral sweep and cautery in the lateral stalk area as well as along the anterolateral prostatic neurovascular bundle helps protect them.

### Circumferential Resection Margin

The circumferential resection margin is the dominant pathologic predictor of pelvic relapse and survival.^50^ A Circumferential resection margin ≤ 1 mm is considered positive across guidelines and carries high risk of local failure. High-resolution MRI reliably anticipates threatened CRM (≤ 2 mm to mesorectal fascia) and should trigger neoadjuvant therapy dedicated to margin sterilization and tumor regression to allow for greater Circumferential resection margin.^40^ All guidelines define CRM positivity as ≤ 1 mm. In population-based data, positive CRM is strongly associated with local and distant recurrence as well as shorter survival.^51^

Oncologic priority is achieving R0 resection, sometimes requiring extended procedures such as extralevator abdominoperineal resection or multivisceral resection (e.g., beyond TME surgery or pelvic exenteration). For anterior T4b disease involving prostate, vagina, or uterus, combined organ resection may be necessary. Lateral sidewall invasion remains challenging, potentially requiring extended resections incorporating internal iliac structures in some cases. While the specific resection needed will vary with each case, the general principle is that en bloc resection of involved structures should be performed. If final pathology reveals a positive CRM, consideration of additional therapy, including postoperative chemoradiation if not delivered preoperatively, should be considered. In cases in which margins are expected to be threatened despite extended resection, intraoperative radiation therapy (IORT) may be considered. IORT has been shown in some retrospective series to be of benefit to patients with R1 resections.^52,53^ Despite the technical challenges of resecting these locally advanced tumors, experienced centers around the world have demonstrated the ability to achieve R0 resections after pelvic exenteration in > 80% and with good survival.

### Distal Margin

Current evidence supports distal resection margins of 2 cm for mid- and lower-rectal cancers assuming TME. For upper rectal cancers, a 5 cm distal margin with a tumor-specific TME is appropriate. Modern guidelines generally consider a 1 cm distal margin to be acceptable for low rectal cancers if it will facilitate sphincter preservation, and 2 cm for more proximal rectal tumors, as long as the CRM is clear. NCCN, ESMO, and JSCCR all accept a 1 cm distal margin in low tumors if necessary for sphincter preservation.^11,54,55^

The choice between sphincter preservation and abdominoperineal resection should be driven by margin reliability and functional considerations. Resection should be defined on the basis of pretreatment imaging, such that tumors that involve the sphincter complex prior to neoadjuvant therapy should undergo Abdominoperinal resection if there is residual disease following neoadjuvant therapy, regardless of whether the tumor has retracted away from the sphincter muscles. In instances in which a distal tumor is felt to be resectable with sphincter preservation, intersphincteric resection allows ultra-low coloanal anastomoses when the external sphincter and levators are intact. When an oncologically sound distal or radial margin cannot be assured, Abdominoperinal resection is indicated, with the universal priority remaining a clear margin over sphincter salvage.

### Lateral Pelvic Lymph Nodes

Rectal cancers below the peritoneal reflection have dual lymphatic drainage pathways, including the superior rectal lymph nodes and the lateral pelvic lymph nodes via the lymphatics along the middle rectal vessels. Management of lateral pelvic lymph nodes illustrates a historical East–West divergence. Western guidelines (NCCN/ESMO) do not advocate prophylactic lateral pelvic lymph node dissection (LPLND), recommending instead chemoradiation to sterilize microscopic disease and reserving selective dissection for nodes that remain radiographically enlarged (typically > 5–7 mm) after neoadjuvant therapy. In contrast, JSCCR defines lateral nodes as regional for low rectal tumors and has historically recommended routine bilateral LPLND for cT3–T4 disease below the peritoneal reflection, noting a higher rate of lateral recurrence, following TME alone, in JCOG 0212.^56^ When performed, LPLND targets the internal iliac and obturator basins with attention to nerve and vessel preservation; while it decreases lateral recurrence, the tradeoffs include risk for increased blood loss and potential autonomic nerve injury. However, for patients with clinical evidence of lateral pelvic lymph node metastasis, neither CRT nor LPLND alone appear to be sufficient for local disease control, and Eastern and Western practices are coalescing around a selective strategy with the growing adoption of TNT internationally, with neoadjuvant therapy followed by lateral pelvic lymph node dissection to achieve surgical clearance of lateral nodal disease.^57,58^

In our practice, patients with clinical evidence of lateral pelvic lymph node metastasis undergo TNT and LPLND of the involved side. Selective node excision is not advised as it leaves potential disease behind and subsequent attempts at LPNLD are associated with elevated risk of nerve and vascular injury.

### Malignant Rectal Polyps

Guidelines generally agree on pathological risk factors that mandate more aggressive treatment. For polyps with unfavorable histologic features (poor differentiation (grade 3/4), lymphovascular invasion (LVI), positive or < 1 mm resection margin, or significant submucosal invasion), there is a substantial risk of residual disease or lymph node metastasis, and surgical resection is recommended.^59^ All major guidelines concur that in a malignant polyp with unfavorable histology, completion radical resection (segmental colectomy for colon polyps, or TME for rectal polyps) should be performed.

For malignant polyps with favorable histology, polypectomy alone can be curative, and careful observation is an option. The NCCN considers polypectomy alone adequate for such low-risk T1 cancers. The risk of lymph node involvement in low-risk T1 cancers is low (~3% or less), whereas high-risk features raise that risk to 15–20%, which justifies an oncologic resection with lymphadenectomy. Transanal local excision can be performed to obtain a full-thickness excision of the scar or residual polyp site to more accurately assess margins and depth.

For T1 rectal cancers that were not removed endoscopically (e.g., visible on endoscopy or biopsy-proven early tumor), the management is similar: if the lesion is amenable to local excision (generally ≤ 3 cm, confined to submucosa, well–moderate differentiation, no lymphovascular invasion on biopsy), a local transanal excision can be performed with curative intent. The excision should be full thickness through the rectal wall into perirectal fat to get an adequate deep margin. If the pathology after local excision shows high-risk features (poor grade, LVI, positive margins, deep invasion into submucosa beyond sm1), then a completion TME surgery is strongly recommended due to risk of nodal metastasis.

For patients managed with local excision alone, NCCN guidelines recommend proctoscopy every 3–6 months for the first 2 years, then every 6 months for a total of 6 years in addition to full colonoscopy at 1 year following excision.^54^

It should be noted that LE is associated with poorer disease-free survival compared with TME.^60^ In one series, the first site of failure following local excision was distant in 38% of cases.^61^ In addition, salvage surgery following local excision is associated with more extensive resection, higher permanent ostomy rate, greater morbidity.^61–63^ Taken together, local excision for confirmed rectal cancer should be offered only in carefully selected patients at centers with significant expertise in this approach.

For T2 cancers, local excision alone is generally insufficient as nodal metastasis occurs in ~20% of T2 lesions.^64^ However, in patients who are medically inoperable or refuse radical surgery, neoadjuvant chemoradiotherapy may be considered with our without local excision. This approach was studied in the ACOSOG Z6041 trial, which showed that chemoradiation followed by transanal excision for T2 tumors resulted in moderate pCR rates and some long-term disease-free survivors.^65^ Guidelines such as NCCN and ASCRS suggest that for a high-risk surgical candidate with a T2 tumor, one could consider chemoradiation with assessment of response followed by watch and wait for cCR patients or local excision as an alternative, with careful counseling of an elevated risk of local recurrence.

All patients managed by local excision or polypectomy should undergo close surveillance because if recurrence is detected early, a salvage TME can still be curative. Surveillance typically includes frequent proctoscopy (for rectal cases), periodic imaging, and CEA monitoring as per guidelines.

**Table:** Guideline Comparisons and Areas of Divergence in Rectal Cancer

Guideline	NCCN	ESMO	JSCCR
Lateral pelvic LN dissection	Not routine. Perform LPLND if clinically suspected lateral nodes persist after neoadjuvant chemoradiation.	Not routine. Perform LPLND if clinically suspected lateral nodes persist after neoadjuvant chemoradiation.	Routine for low rectal T3–T4 tumors (lower border of tumor distal to peritoneal reflection).
Distal margin guidelines	Aim for ≥ 1–2 cm distal margin. A 1-cm margin is acceptable for very low tumors to allow sphincter preservation. If < 1 cm would result, prefer Abdominoperinal resection.	Recommends 2 cm margin for tumors > 5 cm from anal verge, and ≥ 1 cm for tumors in distal 5 cm. Accepts 1 cm distal margin in low rectum to avoid unnecessary APR.	Aim for ≥ 2 cm distal margin. Modern practice accepts ~1 cm if tumor is very low and surgeons perform intersphincteric resection to achieve this.
local excision of early tumors	Permissible for T1N0 with favorable histology (G1-2, no LVI), either via polypectomy or transanal excision. High-risk T1 or any T2 should get TME or additional therapy.	Permissible for T1N0 with favorable histology. Allows local excision after chemoradiation for small T2 in select cases (organ preservation attempt).	Endoscopic resection for favorable T1; recommends surgery for unfavorable T1 or deeper lesions.
Adjuvant therapy	After neoadjuvant CRT + TME: recommends ~4 months of adjuvant chemo for stage III and high-risk II (e.g., FOLFOX). If TNT given additional postop chemo may be omitted for pCR or low-risk cases.	Recommends adjuvant oxaliplatin-based chemo for *all* ypStage III and “high-risk” ypStage II.	Recommends adjuvant chemotherapy for stage III and high-risk stage II routinely. Common regimens include oral fluoropyrimidines or CAPOX.
Nonoperative management for patients with complete clinical response (*watch and wait*)	Supported as an option in select cases with expert surveillance protocols.	Supported as an option in select cases of low rectal cancer with expert surveillance protocols. Also recommends considering less radical approaches for complete or near-complete responders—including local excision.	Not recommended at present. JSCCR explicitly advises against routine nonoperative management outside of clinical studies.
